# It is time to authenticate the Microbiome Sciences with accredited educational programs and departments

**DOI:** 10.1371/journal.pbio.3002420

**Published:** 2023-12-07

**Authors:** Nichole Ginnan, Seth R. Bordenstein

**Affiliations:** 1 The One Health Microbiome Center, Huck Institutes of the Life Sciences, The Pennsylvania State University, University Park, Pennsylvania, United States of America; 2 Department of Biology, The Pennsylvania State University, University Park, Pennsylvania, United States of America; 3 Department of Entomology, The Pennsylvania State University, University Park, Pennsylvania, United States of America

## Abstract

The Microbiome Sciences are at a crucial maturation stage. This Perspective argues that the Microbiome Sciences should now be seen as a flourishing and autonomous discipline and that specialist degree programs and departments should be created to foster continued growth.

## Teaser

The Microbiome Sciences are at a crucial maturation stage. Scientists and educators should now view the Microbiome Sciences as a flourishing and autonomous discipline, creating degree programs and departments that are conducive to cohesive growth.

The first two decades of the 2000s were a defining developmental period for the Microbiome Sciences [1,2]. Technological innovations in profiling, from molecules to ecosystems, unlocked microbial communities and revealed both them and their products to be ubiquitous and influential to the foundation of the biosphere [3–6]. While this early phase produced classic literature for trainees to study, the growth of the Microbiome Sciences has largely occurred within siloed disciplines. For example, microbiome scientists studying plants, oceans, or genome evolution remain largely categorized as botanists, marine biologists, and evolutionary biologists.

Due to its disjointed origins, the Microbiome Sciences educational pipeline faces challenges in the coming decade. Most notably, it lacks autonomy as a certified discipline within the life sciences, missing formal degrees, curriculum concentrations, and cross-systems knowledge. Thus, there is an imperative to train interdisciplinary researchers with the confidence to state, “I am a microbiome scientist”, first and foremost, who have the ability to understand and work across multiple fields. This will provide Microbiome Sciences with the autonomy it earned over the past two decades and now requires to formalize and establish fundamental rules, standards, and applications.

A few new efforts are guiding microbiome education towards a more systematized path. Microbiome research-focused centers and initiatives worldwide have emerged to train their communities through workshops, hands-on training, and conferences. Additionally, small-scale graduate certificates and Master’s-level educational developments have arisen, including: Oregon State University’s microbiome analytics curricula training track in the Microbiology Masters (non-thesis) Program; University of Florida’s and Colorado State University’s graduate certifications in Microbiome and Health and Microbiome Sciences and Engineering, respectively; and King’s College London’s specialized Masters-level degree in Microbiome in Health & Disease.

Intermediate, yet avant-garde designs for tackling the disjointed nature of this discipline include the Max Planck Institute’s Department of Microbiome Sciences in Tübingen and The Pennsylvania State University’s Microbiome Sciences Dual Title PhD Degree. The Department of Microbiome Sciences has autonomy and multiple groups focused on human microbiome research, although it does not grant formal degrees. Students trained in the department participate in the International Max Planck Research School (IMPRS) “From Molecules to Organisms” program and ultimately receive a *Doctor rerum naturalium* (PhD equivalent) from the University of Tübingen that features the IMPRS program, rather than Microbiome Sciences. Conversely, the Microbiome Sciences Dual Title Degree at The Pennsylvania State University allows graduate students to earn the first accredited PhD in the Microbiome Sciences alongside another subdiscipline (e.g., Plant Pathology, Anthropology, Ecology). While the Dual Title helps centralize Microbiome Sciences curricula and training across departments and aids in establishing a baseline standard for microbiome education and research, it does not have an autonomous department or program.

To the best of our knowledge, there are no formal Bachelor’s-level training programs or pre-college full-length courses in microbiomes. However, shorter project-based curricula have made crucial steps towards educational transformation at these academic levels, including Discover the Microbes Within!, Tiny Earth, SEA-PHAGES, and EvolvingSTEM. The current training options listed above are arguably transitional and evolutionary, not full solutions. We propose the following actions to advance accreditation forward (Box 1).

**Box 1. Proposed actions to authenticate accreditation of the Microbiome Sciences**

1. Develop Microbiome Sciences PhD and Master’s degree programs.
2. Establish Microbiome Sciences Bachelor’s degree programs.
3. Organize degree-granting departments.
4. Offer full-term, pre-college microbiome courses and labs.
5. Update biology textbooks to include and integrate the Microbiome Sciences.

First, intercollege or interdepartmental Microbiome Sciences degree-granting programs should be founded, initially at the PhD and Master’s levels and then at the Bachelor’s level, potentially organizing into independent departments in the future. Continued, robust support for microbiome research from funding agencies will help drive the field’s sustainability and these degree efforts forward. The same path proved successful for the genomics field that now has dedicated academic departments in the basic sciences, hospitals, and governmental agencies [7,8]. With an academic degree directly reflecting coursework and skills, microbiome scientists will emerge with credentials front and center as they seek out a booming job market in academia and industry. Indeed, there is a growing workforce need for the microbiome biotechnology market that is expected to reach a $300 billion USD value by the end of the decade [9].

Second, full-term Microbiome Sciences courses and labs tied to degree-granting programs should be explicitly established at pre-college levels, accompanied by new basic biology and microbiome-specific textbooks that devote substantial chapters and integration to existing sections on visible life. Content should span the ubiquitous nature of microbes, vital functions within and across ecosystems, and hands-on technology labs that peek into the hidden microbial world with culturing, sequencing, etc. Designing and implementing microbiome-specific, pre-college courses will alter students’ and eventually the public’s awareness, overturning a century of precedent and emphasis on visible life, with the reality that life invisible to the eye is dominant by all measures [10]. Students ought to be taught and trained from this reality. In the next decade, holistic microbiome-educated thinkers will enter non-research fields (medical school, nursing, engineering, urban planning, policy making, etc.), and this level of integration of microbial knowledge into society may facilitate an aspirational utopia with microbiome-informed consumers, legislators, and providers.

Traditionalists may ask, “… is Microbiome Sciences just Microbiology, Ecology, or Genomics?” The answer is no. It does not simply fit into any one of those boxes, but rather it is the nexus of those and several other fields, thereby making it a unique synthesis—an intricate composite of field, wet lab, theoretical, and computational work aimed at decoding microbial interactions with hosts and with each other [11,12]. Similarly, microbiome researchers often experience a limbo in expertise, relating to the Shakespearean phrase, “a jack of all trades, a master of none.” We ought to validate these individuals as expert Microbiome Scientists with ratified educational opportunities at every academic level.

As this robust discipline continues its rapid growth and spawns novel focus areas (including gnotobiotics (microbe-free systems), synthetic microbial community engineering, holobiont biology, and high-throughput profiling of organismal DNA, RNA, proteins, and metabolites), it is imperative that the next steps lead to validation of the Microbiome Sciences as an autonomous field through the development of degree-granting programs and, possibly, of independent educational and research departments. The origin-to-ratification story for the genome sciences occurred after its first two decades; now is the time for it to materialize for the Microbiome Sciences. The best students in the discipline will undoubtedly seek out these programs.
